# Photodynamic Therapy: From the Basics to the Current Progress of *N*-Heterocyclic-Bearing Dyes as Effective Photosensitizers

**DOI:** 10.3390/molecules28135092

**Published:** 2023-06-29

**Authors:** Eurico Lima, Lucinda V. Reis

**Affiliations:** 1 CQ-VR—Chemistry Centre of Vila Real, University of Trás-os-Montes and Alto Douro, Quinta de Prados, 5001-801 Vila Real, Portugal; eurico_lima@icloud.com; 2 CICS-UBI—Health Sciences Research Centre, University of Beira Interior, Av. Infante D. Henrique, 6201-506 Covilhã, Portugal

**Keywords:** *N*-heterocyclic-based dyes, photosensitizers, photodynamic therapy

## Abstract

Photodynamic therapy, an alternative that has gained weight and popularity compared to current conventional therapies in the treatment of cancer, is a minimally invasive therapeutic strategy that generally results from the simultaneous action of three factors: a molecule with high sensitivity to light, the photosensitizer, molecular oxygen in the triplet state, and light energy. There is much to be said about each of these three elements; however, the efficacy of the photosensitizer is the most determining factor for the success of this therapeutic modality. Porphyrins, chlorins, phthalocyanines, boron-dipyrromethenes, and cyanines are some of the *N*-heterocycle-bearing dyes’ classes with high biological promise. In this review, a concise approach is taken to these and other families of potential photosensitizers and the molecular modifications that have recently appeared in the literature within the scope of their photodynamic application, as well as how these compounds and their formulations may eventually overcome the deficiencies of the molecules currently clinically used and revolutionize the therapies to eradicate or delay the growth of tumor cells.

## 1. Introduction

Photodynamic therapy (PDT) is a minimally invasive therapeutic strategy that intends to be applied to the most diverse problems of our society’s daily lives: as an anticancer therapy [1,2,3], as a way of combating multi-resistant bacteria [4] or certain fungi [5] that may compromise human health, and, more recently, as a way of eradicating certain viral strains [6,7]. PDT already plays a role in treating several non-oncological and oncological diseases, mainly of dermatological origin [8]. Its slow clinical progression in cancer treatment is primarily due to the lack of approved photosensitizers (PSs). Nevertheless, researchers have tried to find new attractive molecules and their formulations that perform better than those currently used.

Focusing on cancer, this term refers to a wide range of diseases that trigger abnormal growth and division due to cell proliferation and regulation mechanism modifications. Given their uncontrolled behavior, they become highly invasive and can even reach the bloodstream and migrate to places where extravasation allows the formation of metastases [9]. In addition to environmental and hereditary factors, lifestyle, and exposure to carcinogens, aging is one of the factors that most contribute to the onset of this disease [10,11]. Although the scientific, pharmaceutical, and medical communities have made efforts to improve the available treatments, the long-term response of patients treated with radiotherapy and chemotherapy is still insufficient due to the severe side effects [12,13]. These gaps highlight the need to explore emerging therapies such as PDT. Clinical studies have shown that this therapeutic modality can be curative, especially in less advanced tumors [14,15]. In addition, it can prolong survival in cases of inoperable tumors and improve patients’ quality of life [3,15].

Featuring heterocyclic units accompanied by nitrogen atoms in their molecular structure as well as conjugated double bonds that allow the design of chromophores, *N*-heterocyclic-bearing dyes constitute several families of compounds that, thanks to their relevant “photoproperties”, have been addressed concerning their potential application as PDT photosensitizing agents. Hundreds of new synthetic or hemisynthetic origin molecules have been reported in recent years to find the compound that combines desirable characteristics for its phototherapeutic application and better understand how and why certain structural modifications result in such diverse photobiological effects.

**Figure 1:**
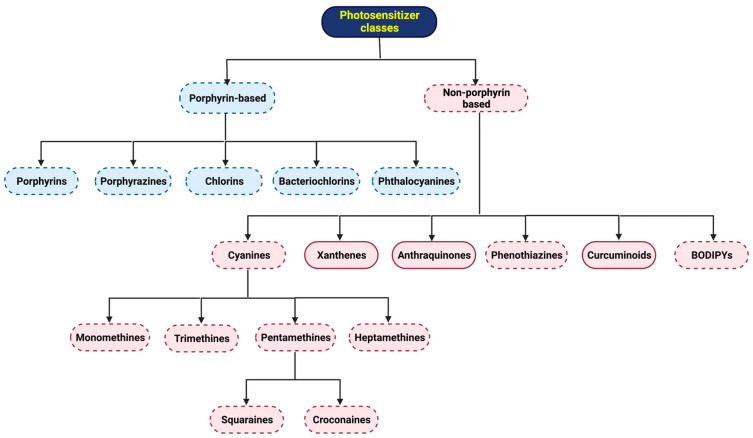
Flowchart hierarchy of the photosensitizer classes currently studied. The categories and respective sub-categories whose box limits are in dashed lines are related to dyes with *N*-heterocyclic units in their basic structure.

This review aims to present the reader with a concise approach to what PDT is and its fundamental principles, its importance as a therapeutic approach to complement or even replace conventional therapies in the treatment of diseases of oncological origin, several parameters to take into account to guarantee better efficiency and efficacy, as well as some of the families of *N*-heterocyclic-bearing dyes and the structural modifications or their formulation strategies that have been recently carried out to create new light-absorbing potential drugs with photodynamic interest (Figure 1).

## 2. Photodynamic Therapy

### 2.1. A Piece of Photodynamic Therapy’s Origin and History

Dating back to 1550 B.C., the Ebers papyrus is the oldest medical document that reports the use of sunlight as a treatment, using powder from various plants such as bishop’s flower (*Ammi majus*), wild parsnip (*Pastinaca sativa*), parsley (*Petroselinum crispum*), and St. John’s wort (*Hypericum perforatum*) for the application and treatment of depigmented skin lesions [16]. Also in Egyptian culture, pharaohs called for the construction of roofless temples dedicated to the Aton god of the sun for body light exposure and to benefit from its healing powers, and the use of *Ammi majus* plant extracts was common in the treatment of diseases of a dermatological nature [17]. Indian medical literature dated 1400 BC, the Atharva Veda, reports treating vitiliginous skin with babchi (*Psoralea corylifolia*) extracts, a plant from which it is now known that 8-methoxypsoralen is the furan-containing coumarin-based molecule that induced the photodynamic effect [16,18]. Even today, this core of photosensitizing molecules is widely studied for its potential phototherapeutic use [19,20]. Also, in Greek, Chinese, and Roman civilizations, there is evidence of the use of energy from the sun as a remedy for the treatment of various diseases.

Arnold Rikli, a Swiss healer and physician, reintroduced the use of the powers of sunlight during the late 1800s and early 1900s. A believer that nature was the basis for treating many of the complications related to human health (mentioning his famous phrase, “Water is good; air is better and light is the best of all”), Rikli trusted in the power of light in therapy, introducing “sunbathing” as a therapy for chronic diseases and functional disorders in an attempt to apply the knowledge that had been known for hundreds of years but had been forgotten [16,21].

Until then, the effectiveness of using sunlight to treat various diseases had been proven, namely for vitiligo, lupus, acne, pulmonary tuberculosis, and rickets, using (or not) extracts and powders from plant species administered topically or orally. However, it was only in 1900 that the concept of a “photosensitizing molecule” emerged, with the potential use of dyes in this therapy having been recognized: Oscar Raab, a medical student, reported that the light factor was lethal for species of *Paramecium caudatum* in the presence of the acridine dye and that these two elements, individually, did not cause any consequences to this unicellular organism [22]. Taking advantage of these discoveries, von Tappeiner continued Raab’s research with the dermatologist Jesionek, who published works detailing clinical trials using dyes such as eosin, fluorescein, and sodium dichloroanthracene disulfonate in the treatment of topical disorders, including skin cancer [23,24]. At the same time, they also reported data on the possible use of eosin in facial basal cell carcinoma after sun exposure or using light from arc lamps. Von Tappeiner was the first to speculate about the need for triplet molecular oxygen, introducing the term “photodynamic therapy” [16].

Despite the significant findings of Raab and von Tappeiner, Niels Finsen, a Danish physician, is considered by many to be the pioneer of modern clinical PDT. Finsen owned a medical institute in Copenhagen, to which he attached a “sun garden” to allow his patients to benefit from the healing powers of sunlight. Later, he abandoned the sunlight to start using artificial lights and noticed that exposure to red or ultraviolet light could have therapeutic effects since red light prevented the formation of pustules in patients with smallpox and that ultraviolet light allowed the treatment of cutaneous tuberculosis [16,23,25]. Due to the impact of his discoveries, Finsen was awarded the Medicine Nobel Prize in 1903.

A noteworthy milestone demonstrating the acceptance of PDT as a therapeutic modality was the approval of Photofrin in 1993 for treating bladder tumors in Canada and later in the United States, Japan, and some European countries [26,27]. Today, the Photofrin oligomer is used worldwide in treating oesophageal, endobronchial, and lung cancer, in addition to being used in Japan for gastric cancer. Since its discovery, new molecules have been clinically approved, namely 5-aminolevulinic acid and methyl 5-aminolevulinate (Levulan and Metvix, respectively), precursors of porphyrins, in the treatment of basal cell carcinoma and squamous cell carcinoma; in Japan, Talaporfin chlorin (also known as Laserphyrin) in the treatment of glioblastoma and lung cancer; in Russia, the chlorin mixture Radachlorin in basal cell carcinoma and the sulfonated phthalocyanine Photosens for lung, liver, breast, skin and gastrointestinal cancer; and in the European Union, Foscan chlorin and Redaporfin bacteriochlorin have gained a role in the therapy of head and neck cancer, and Tookad bacteriochlorin in prostate cancer [28].

However, from then on, there was a growing need to synthesize new molecules with greater efficacy and fewer side effects, giving rise to new “second- and third-generation PSs” [29,30].

**Figure 2:**
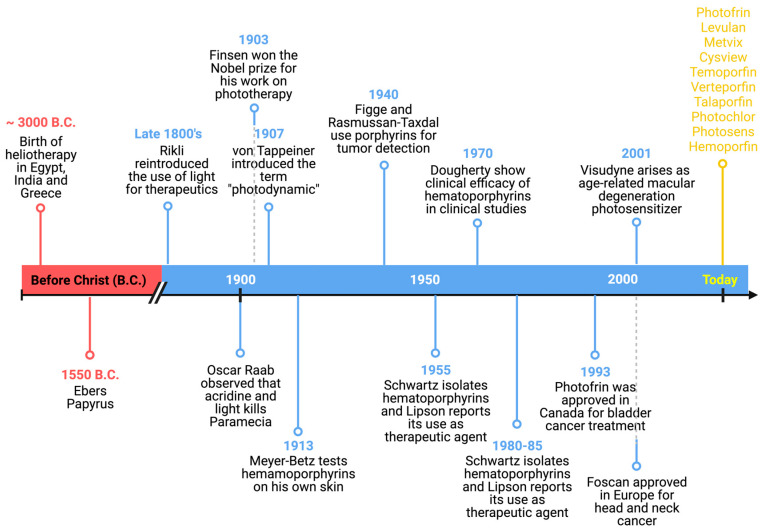
Timeline of photodynamic therapy, highlighting some of the events that most marked its evolution up to the present day.

These and other milestones in the history and progress of PDT to date are highlighted in the timeline in Figure 2.

### 2.2. Principles of Photodynamic Therapy

Clinically, the PS is administered through the most convenient route, depending on its pharmacokinetic and pharmacodynamic properties and the location of the target tissue. When administered intravenously, the photosensitizing agent is distributed throughout the body, ideally directing and accumulating selectively in the tissue to be treated. At the time of administration, the patient must be deprived of any light source to avoid premature activation. Retained by the target tissue and before being eliminated from the body, the same tissue is irradiated using a suitable light source, preferably emitting where radiation permeates tissues more efficiently, at wavelengths coincident with the molecule absorption, and with sufficient energy for its activation. Activation of the compound culminates in cell death and consequent ablation of the target tissue [3,31,32].

According to how the photosensitizing molecule acts, in general, it can produce reactive oxygen species (ROS) [33] that, despite being essential for the normal functioning of the cell [34,35], in high concentrations, become cytotoxic [34]. Thus, this therapeutic approach results from combining three individually innocuous components that are lethal when combined: the PS, light, and triplet state oxygen molecules.

**Figure 3:**
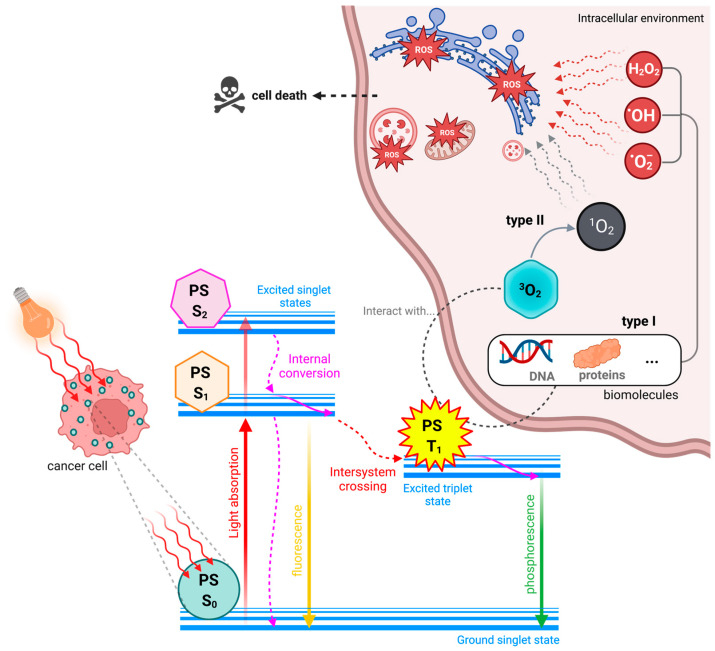
Photodynamic therapy-customized Perrin–Jablonski energy diagram. After cellular uptake of the photosensitizer (PS) and irradiation with light emitting a specific wavelength, this radiation is absorbed by the PS in the ground state (PS S_0_), which transitions to the first or second excited singlet state (PS S_1_ and PS S_2_, respectively). Variations between PS S_1_ and PS S_2_ or between the various energy levels of the same state occur by internal conversion. Radiative (fluorescence and consequent return to S_0_) or nonradiative (intersystem crossing to the PS T_1_) processes can occur in the PS S_1_. The PS T_1_ can interact with biological substrates, forming reactive oxygen species (ROS), such as hydrogen peroxide (H_2_O_2_), hydroxyl radical (^•^OH), or superoxide anion (^•^O_2_^−^), by type I reactions, or singlet oxygen (^1^O_2_) by type II reactions. In high concentrations, these ROS led to cell damage in essential structures for normal cell functioning, culminating in cell death.

The Perrin-Jablonski energy diagram explains photophysically and photochemically the activity of PS compounds (Figure 3). This diagram elucidates that these molecules, in their ground state, are in the lowest possible energy configuration with opposite spins. When appropriate radiation is applied, an electron is transferred to a higher energy state, transiting the PS to the excited singlet state, a highly unstable energy configuration that easily returns to its fundamental state, emitting a photon in the form of fluorescence. The ground state PS does not result in therapeutic activity; however, the ability of dyes to fluoresce can be harnessed to create diagnostic or even theranostic techniques. Only if the PS transitions from the excited singlet state to the excited triplet state by intersystem crossing (ISC), that is, the inversion of the excited electron spin, is the drug capable of carrying out photodynamic reactions (of type I or type II). The loss of energy resulting from the transition of the PS from the triplet state to the ground state occurs through phosphorescence emission [1,31].

Type I reactions are characterized by the interaction of the triplet excited state PS with cellular substrates and the consequent formation of free radicals through the transfer of electrons or protons to form anion or cation substrates, respectively [36,37]. An electron transfer from these radical substrates to molecular oxygen in the triplet state results in superoxide anions, which can produce hydrogen peroxide when reacting with water. If the production of hydrogen peroxide and superoxide anions is exacerbated, highly cytotoxic hydroxyl radicals may be formed through the Haber–Weiss reaction. Hydroxyl radicals can also be produced in the presence of, for example, iron or copper metal ions by the well-known Fenton reaction. The production of these radicals is desirable for photodynamic activity due to their distinct reactivity and ability to oxidize any molecule of biological interest [38,39].

Type II reactions are responsible for forming singlet oxygen through direct energy transfer between the triplet state PS and the triplet state molecular oxygen. Despite its high cytotoxicity, singlet oxygen has a minimal lifetime, so its action is mainly limited to the place of its generation [40]. Type I and type II reactions can coexist, and the prevalence of one type over the other is mainly related to the intrinsic properties of the PS [41]. Other conditioning factors are how the PS is distributed in the cellular environment, its proximity to biological substrates, and the triplet molecular oxygen concentration around it.

**Figure 4:**
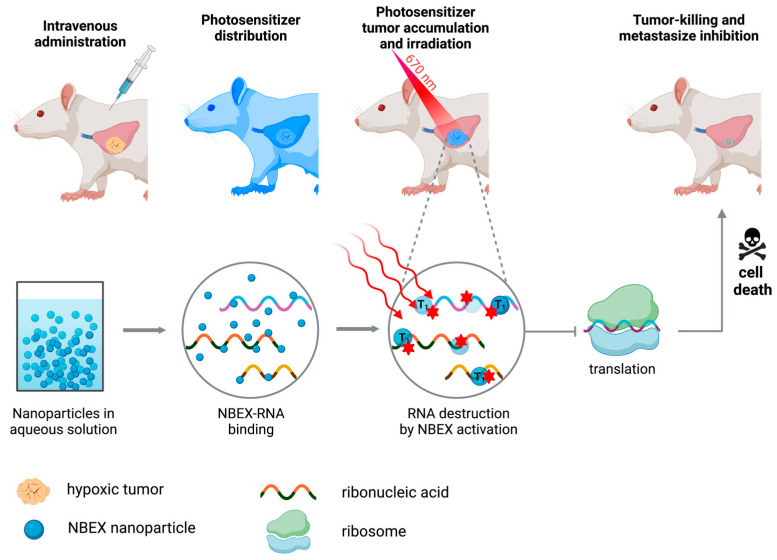
Photodynamic action mechanism of ribonucleic acid (RNA)-targeting self-assembling NBEX dyes type III-photosensitizer prepared by Yao *et al.* [43]. Nanoparticles were prepared in an aqueous medium by self-assembly of NBEX dyes. After administration, distribution, and accumulation in the target tissue, it was irradiated using a light source at 670 nm. At that moment, the NBEX, found linked to RNA molecules of interest for normal cell functioning and maintenance, were destroyed by the photosensitizer in the triplet state, regardless of the coexistence of molecular oxygen. As no RNA molecules are available for the regular production of proteins essential to cell integrity, the death process is triggered, resulting in the eradication of the tumor.

Photosensitizing molecules capable of tissue destruction through mechanisms independent of the presence of molecular oxygen in the triplet state (type III reactions) have also been reported. These type III PSs have the intrinsic property of specific targeting for biomolecules, such as proteins and nucleic acids, among other biomacromolecules. As a result, these target biological elements are efficiently destroyed when irradiated, compromising normal functioning and survival [42]. Recently, new type III *N*-heterocyclic dyes called “NBEX” were reported, which, after self-assembling and forming NBEX nanoparticles, bind specifically to ribonucleic acid (RNA) molecules, destroying them after irradiation and inhibiting the normal synthesis of proteins essential for vital cellular processes [43] (Figure 4).

### 2.3. Photodynamic Therapy Clinical Applications

The clinical application of PDT began in dermatology. In this medical specialty, its purpose is much easier since drugs and their formulations can be administered topically, and access to the lesion is facilitated [44]. In addition, given the restricted penetration of light into biological tissues and the difficulty in delivering the energy required for PS activation to deeper tissues [45], the cutaneous surface is an organ of excellence concerning phototherapeutics. Photodynamic treatments in dermatology have shown promising results, with aesthetic improvements and similar therapeutic outcomes to surgery or radiotherapy in treating actinic keratosis, Bowen’s disease, and basal cell carcinoma [46]. This reinforces the need to expand PDT into other areas of medicine.

As such, strategies were gradually created for treating diseases of various medical natures, such as in the field of ophthalmology, in the fight against age-related macular degeneration, pachychoroid disease, and oncological disorders such as choroidal hemangioma, and retinal hemangioblastoma [47,48]; urology, in the treatment of bladder, prostate, urethra, ureter, penis and kidney malignancies [49,50]; otorhinolaryngology, in the control of tumors in the head and neck [51,52], including carcinomas of the mouth, pharynx, larynx and ear; gynecology, in the treatment of carcinoma of the cervix, vulva and vagina [53,54,55]; and being applied to tissues of the gastrointestinal tract, such as in the therapy of high-grade Barrett’s esophagus and esophageal cancer [56,57,58].

Despite being recognized worldwide for their comparative promise with conventional strategies, all potential drugs, before being clinically applied, must undergo clinical trials that prove their safety, effectiveness, and possible side effects in the short and long term. For PDT, clinical approval is difficult, as it is necessary to consider not only the therapeutic properties of the PS but also its combination with an energy radiation application system and its light dosimetry.

### 2.4. Photodynamic Therapy Advantages and Disadvantages Compared to Other Strategies

Chemotherapy, a therapeutic strategy based on the systemic administration of a cytotoxic product, despite allowing it to reach tumor cells in different parts of the body and efficiently eradicate them, has the most significant disadvantage of causing damage to healthy cells [59]. Furthermore, numerous side effects have been observed depending on the chemotherapeutic agent used, such as nephrotoxicity, hepatotoxicity, ototoxicity, neurotoxicity, cytopenia (leukopenia, neutropenia, anemia, and thrombocytopenia), nausea, vomiting, and alopecia [60]. Similarly, radiotherapy, in addition to the numerous adverse effects it causes in patients (fatigue, hair loss, damage to the epidermal layer, swelling, loss of memory and tenderness, fertility issues, and cardiovascular complications) [61], is unable to induce death in tumor cells that cannot be seen in imaging exams [62], is ineffective in the treatment of tumors with little blood supply [63], and needs to be administered for a continuous period of 1 to 2 months for best results [64].

To improve these negative features, research has been guided toward searching for more viable, safer, and more successful alternatives for treating cancer. Photodynamic therapy has precisely this objective, offering advantages such as:

- Minimal invasiveness: The PS administration is carried out intravenously, intratumorally, or topically, followed by irradiation to the surface or light delivery performed through endoscopy techniques, avoiding surgical strategies [3,65];
- Double selectivity: It is spatially selective, as the light beam is focused on the precise location of the target tissue, and, as the PS preferentially accumulates and retains at the site to be treated, systemic toxicity is minimized [66]. Furthermore, as the half-life of the ROS produced, such as singlet oxygen and hydroxyl radicals, is less than a microsecond, reactions of an oxidative nature hardly occur in adjacent healthy tissues;
- Combinable with other therapies: If PDT does not produce the desired effect, it can be used in combination with other therapeutic modalities, such as chemotherapy, radiotherapy, surgery, and immunotherapy, among other more invasive means, since drug interactions do not occur as therapeutic targets are different from those of PDT, and irradiation treatment does not interfere with the activity of other antineoplastics [67,68]. Therapeutic targets differ, making their combination non-competitive and capable of producing improved effects compared to single treatments;
- Fewer adverse effects: Compared to conventional therapeutic strategies, which cause intensive wear and tear on the patient, PDT has some adverse effects at the cutaneous level, such as pain, burns, erythema, edema, itching, desquamation, and pustular formation [67,69];
- High effectiveness: At an early stage and for localized solid tumors, PDT can be a very effective treatment. In the case of patients in a palliative state, this therapeutic modality can be an alternative to the conventional ones since it can significantly improve the quality of life [70,71];
- Reduced recovery time [72];
- Repeatable in case of tumor recurrence: Photodynamic treatment can be repeated in the event of the appearance of a new primary tumor in a previously treated area without risk of damage to surrounding normal tissues or development of resistance to therapy, mechanisms recurrently observed and reported in the literature regarding conventional medicines, which limit their effectiveness [66,73,74];
- Lead to better aesthetic results: Especially in cases of skin cancer, good aesthetic results are observed compared to surgical methods [75]. Photodynamic treatment should not cause an increase in the temperature of the tissues, causing no destruction of the connective tissue and allowing the anatomical and functional integrity of the tissues to be maintained [76].

On the other hand, and like any therapeutic approach, PDT has several limitations that condition it clinically:

- Few commercially available PSs: Not many PS molecules are approved for clinical use. Furthermore, most PS currently applied are derived from porphyrins, a family of molecules whose molar absorptivity at higher wavelengths, in the regions of the electromagnetic spectrum where tissues are more transparent to light, is relatively low [77];
- Reduced penetration of light into tissues: The wavelengths used in therapy can induce cell death within a maximum radius of 10 mm from the illuminated area, which is often insufficient for eradicating a larger tumor mass [78,79];
- Dependence on the presence of triplet molecular oxygen: PSs whose mechanisms of action are type I and type II reactions depend on triplet molecular oxygen since this is necessary for forming reactive species that induce cell death [80]. Developing new type III PSs could circumvent this limitation [43];
- Absence of contact with light sources for significant periods: Depending on the pharmacokinetic and pharmacodynamic properties of the PS, the time it takes to reach and accumulate preferentially in the target tissue is variable. However, during this period, designated by many as the “drug-light interval”, the patient cannot be exposed to any light, as this would activate the compound prematurely [26,81];
- Persistent cutaneous photosensitivity: Even after photodynamic treatment, the patient should not be exposed directly to light sources since the PS takes some time to be eliminated from the body. From light incidence until the patient can be exposed to radiation, it depends mainly on the photosensitizing molecule. Early exposure results in skin photosensitivity, an effect that may last over time [82];
- Dosimetry is difficult to prescribe because several parameters must be optimized before applying therapy. The PS dose to be administered, the “drug-light interval”, as well as the light source to be used, the irradiation area, and the dose of light to be applied must be determined considering factors such as the size and location of the tumor and the level of oxygenation of the tissues to be eliminated [83].

## 3. The “Three Elements” of Photodynamic Therapy

### 3.1. Light Source

Illumination is a critical element of PDT, as its clinical effectiveness depends on the accuracy of light delivery at an appropriate dose [84]. Thanks to technological advances, delivering light accurately and in adequate amounts to a large part of the human body is possible. Therefore, this therapeutic approach can be applied to a wide range of diseases [78]. The choice of radiation source (Table 1) should depend on the location of the tumor and its depth. However, its dosimetry is still difficult to estimate, including the time of radiation exposure, the fluence of light, and the mode of light delivery (whether continuous or fractionated). Nevertheless, dosimetry is crucial for therapeutic success, knowing that delivering a high dose of light in a short period of time can lead to a rapid depletion of triplet molecular oxygen, compromising the destruction of the target tissue [31,85]. There is evidence that the most viable methodology is to carry out prolonged photodynamic treatments using lower irradiation energies [86,87].

**Figure 5:**
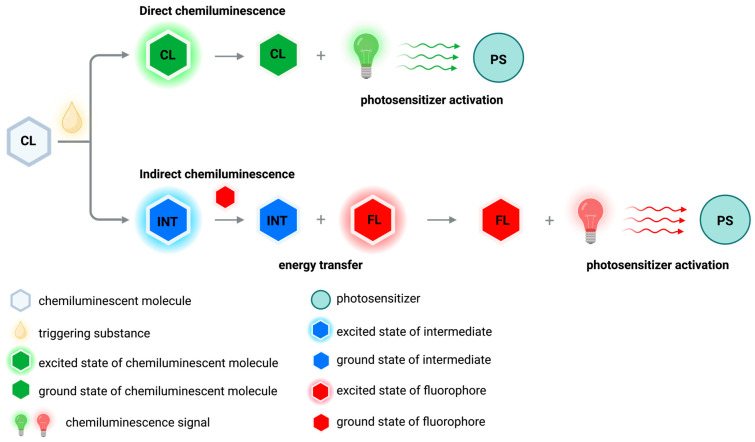
Basic principles of chemiluminescence-mediated photodynamic therapy as an alternative to treatment using external light sources. While in direct chemiluminescence, the excited material (CL) itself is capable of emitting light, during the process of indirect chemiluminescence, the energy is passed from the chemiexcited molecule (the intermediate (INT)) to another molecule (known as the acceptor or fluorophore (FL)) which becomes further excited. The emitted energy is absorbed by the photosensitizer (PS), activating it.

More recently, alternatives to conventional light sources have been studied to create a therapeutic strategy independent of external light that overcomes the fact that light cannot permeate tissues as efficiently as desired [94]. An example of these alternatives is chemiluminescence-mediated PDT (CL-PDT), based on the joint use of the PS and a chemiluminescent molecule [95] (Figure 5). These chemiluminescent molecules can emit energy in the form of radiation directly or indirectly: directly when the chemiluminescent molecule is activated by oxidation, forming a high-energy intermediate that dissipates energy by emitting photons until it returns to the ground state, and indirectly when these intermediates release energy that is subsequently absorbed by nearby fluorophores [96]. Luminol and its derivatives are direct chemiluminescent compounds that emit energy in the presence of oxidizing agents [97]. They were already studied for potential application in PDT [98]. To ensure that luminol irradiates the photosensitizing molecule, unique molecules resulting from the conjugation by covalent bonds of the chemiluminescent agent and PS were recently reported [99].

In addition to visible light, there are several alternatives for activating PSs [100], including microwaves, near-infrared (NIR) and infrared light, x-rays, and ultrasounds [101].

### 3.2. Triplet Molecular Oxygen

Triplet molecular oxygen plays a significant role in the PDT process, and its availability in the target tissue critically affects the treatment outcome when using PSs whose mechanism of action concerns mainly type I or type II reactions.

Since triplet molecular oxygen is essential for therapeutic success, hypoxia conditions can considerably jeopardize the effectiveness of the treatment [102]. One of the consequences inherent to PDT is tumor hypoxia itself since this strategy is based on the consumption of oxygen present in the tissues for its destruction and, in several cases, the vasculature that irrigates it [103]. Furthermore, tissue molecular oxygen concentration can vary significantly between tumors and regions of the same tumor, depending on the density of the vasculature that irrigates it and the rates of diffusion and consumption of oxygen [104]. Thus, irradiation with high-power light sources can be disadvantageous as it leads to total oxygen consumption [86,105].

Monitoring the concentration of oxygen in the tissues is a remarkable tool that allows the adjustment of the power of the incident light so that, ideally, the amount of oxygen consumed is equivalent to that diffused to the tumor tissue to guarantee the continued production of ROS during the period of irradiation without depletion of the component from which they are produced [106,107]. In addition, in tumors of a hypoxic nature, it is predictable that there will be evidence of resistance to therapy when type I or type II PSs are administered [108,109], so the use of PSs that produce type III reactions is desirable in this circumstance [43]. Alternatively, PDT experiments carried out with patients in hyperbaric chambers significantly improves the efficiency of the treatment of tissue ablation [110,111].

### 3.3. Photosensitizer

Thousands of compounds, dyes, and pigments with photosensitizing capacity have already been identified, either of natural [112], synthetic [113], or hemi-synthetic origin [114]. Porphyrins are the most widely studied of the various classes of PSs reported in the literature. However, due to several limitations that these dyes have, such as the low absorption in the red and NIR regions and higher absorption in blue and green, where tissues are less transparent to light, new alternatives have been sought to improve them [115].

Proof that efficient photoreactive molecules are present in nature is that insects that fed on the green fungus *Cortinarius austrovenetus*, after exposure to solar radiation, ended up dying, revealing the presence of compounds with photocytotoxic activity. It was discovered that this is a fungus-specific mechanism: damaged tissues of the green mushroom become violet due to the oxidation of the pigment austrovenetin in hypericin [116,117], one of the most studied PSs today [118]. Nowadays, it is known that, in addition to *Cortinarius austrovenetus*, a greater diversity of plants, such as *Matricaria chamomilla*, *Spinacia oleracea*, and *Aloe vera*, have similar defense strategies [116]. With advances in analytical technology, photosensitizing substances are becoming more easily discovered in natural extracts. As the identification of these “biophotoactive” molecules continues to increase, it is expected that many more will be found in the coming years [119,120]. Therefore, using PSs derived from plants can be considered an eco-friendly method of PDT [120].

Because families of natural compounds exhibit desirable properties for their phototherapeutic application, structural modifications have been carried out to enhance them, make them more biologically active, or give them more attractive photophysicochemical properties [121,122]. In addition to the porphyrins themselves, widely found in nature in a wide variety of organisms, synthetic analogs of pyranoanthocyanines, dyes that form during the maturation of red wine, have also been explored for their photobiological effects with promising results [122].

Regardless of their natural or synthetic nature, the photosensitizing candidate must exhibit specific properties for their phototherapeutic applicability. Table 2 presents the characteristics of an ideal PS for PDT, on which research has focused when looking for new candidates to be applied in this therapeutic strategy. However, it is crucial to remember that finding a PS with all these qualities is difficult. Furthermore, depending on the application, the most efficient PSs will likely compromise these properties.

## 4. *N*-Heterocyclic-Bearing Dyes as Photodynamic Therapy Photosensitizers

**Figure 6:**
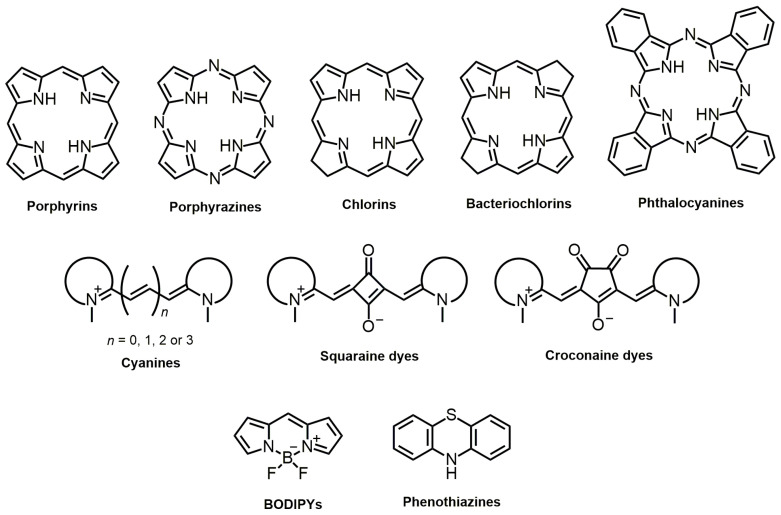
Skeleton structures of *N*-heterocyclic-bearing family dyes addressed in this review article.

In an endless search for molecule structures that combine the most significant number of properties inherent to those of an ideal PS, several dyes have been described in the literature, namely *N*-heterocyclic-bearing dyes (Figure 6). These dyes have the characteristic of having at least one heterocyclic unit in their molecular structure, that is, at least one cyclic structure with at least two different elements, in this case, carbon and nitrogen. Thus, this nucleus of dyes comprises a wide variety of families of compounds of porphyrin- and non-porphyrin nature. Several structural modifications and formulations, including their encapsulation in nanoparticles, have been studied to understand and make known to the scientific community how advantageous they are for their potential phototherapeutic applicability and to create diagnostic or therapeutic approaches.

### 4.1. Most Recent Studies Regarding Photosensitizing Candidates’ Discovery

#### 4.1.1. Porphyrin-Based Photosensitizers

##### Porphyrins

Porphyrins are a class of organic compounds whose chemical structure is composed of a ring formed by four *N*-heterocyclic pyrrole-type units connected by four methine bridges [130]. Its presence in nature and its role in various biological processes are well documented [131,132]. Generally, these dyes family can efficiently absorb visible light, preferentially in the blue and green regions, and produce singlet oxygen and free radicals proficiently [113,133]. However, due to their low solubility in water, short circulation time, and lack of specificity for tumors, traditional porphyrins have limited effectiveness as PDT PSs and diagnostic tools [134,135,136]. To address these shortcomings, scientists have developed various methods to improve the efficiency of porphyrins through chemical modification and nanofunctionalization techniques [137].

Concerning structural modifications carried out in porphyrins, the research focus has been directed to the functionalization of methine protons in the *meso* position by groups of diverse natures and the coordination of free base protons by metallic ions. Additionally, since it is the oldest known class of PSs and given that structure-activity effects are already relatively well known, the preparation of nanoparticles that incorporate these compounds and allow improving specific gaps in these candidates for PSs has been highly reported in recent literature.

The preparation of metalloporphyrins can significantly improve the phototherapeutic effects of analogous free-base porphyrins. For example, very recently, in the study by Hou *et al.* [138], the activity of water-soluble porphyrins coordinated with zinc and copper was compared with that of uncoordinated porphyrins, and it was verified that the one bearing zinc exhibited increased singlet oxygen and peroxide formation. Contrary to zinc, the copper coordinate did not reveal any therapeutic effect.

In addition, several investigators have studied biological effect variations for porphyrins coordinated with other metals such as palladium, manganese, and nickel [139,140,141,142,143]. According to these studies, coordination with palladium was not as advantageous as with platinum [139], and for porphyrin dyes complexed with copper, nickel, and zinc ions, the copper derivative showed the worst photodynamic effects [140]. In contrast to most research involving metalloporphyrins, Frant *et al.* [142] conclude in their work that manganese-metalized porphyrin is, by itself, cytotoxic, while its free-base analog induces effects only when irradiated on colorectal cancer cells. *N*-heterocyclic carbene, gold, *meso*-functionalized zinc, and palladium coordinated porphyrins prepared by Scoditti *et al.* [143] showed no cumulative impact on the ISC rate, revealing that the introduction of metals at different porphyrin positions does not induce a synergistic effect.

Regarding the modifications made to the *meso* porphyrin position, several functional groups have been reported, depending mainly on the therapeutic or functional aim:

- Inferring the functioning of specific cell receptors, Manathanath *et al.* [144] synthesized a series of tetrahydroxiphenyl-derived porphyrins (THPP) appended with the 4,6-diamino-1,3,5-triazine group. This moiety was introduced since triazines are known for their attractive bioactivity due to their kinase receptor inhibitory nature, particularly their ability to inhibit the epidermal growth factor receptor-tyrosine kinase, overexpressed in tumor cells, which is involved in tumor proliferation, metastasis, and angiogenesis processes [145,146];
- Responsiveness to elements overexpressed in the tumor environment: Huang *et al.* [147] designed a “dual response” porphyrin-based PS capable of responding to the typical increase in glutathione (GSH) and hydrogen sulfide concentration in tumor cells. For this purpose, they prepared a reversible derivative of the already known THPP esterified with 2,4-dinitrobenzosulfonyl chloride, which presented low to moderate toxicity and zero formation of singlet oxygen. Furthermore, the investigators testified that only in the presence of GSH and hydrogen sulfide occurs the photosensitizing agent activation by the quenching group departure, stating that it has a high clinical potential in reducing the effect of cutaneous photosensitivity;
- Improvement of functional properties of this family of PSs: Certain functional groups can significantly contribute to refining physiological and chemical stability and biocompatibility [148]. The introduction of polyethylene glycol (PEG) moieties into porphyrin cores, for example, has been reported several times in the literature [149,150,151]. Lazewski *et al.* [151] demonstrated that, irrespective of the coordinated metal, short-PEGylated porphyrins reveal reduced dark cytotoxicity, increased ability to produce singlet oxygen, and that the structural location where the polymer is introduced can result in the variation of the biological effects;
- As an interface between single-PSs and nano-PSs, Li *et al.* [152] demonstrate that the chain conjugation of PEG-bearing porphyrins with perylene diimide units resulted, by self-assembly, in a kind of nanoparticle with attractive properties: (i) high biocompatibility; (ii) intense absorption in the visible and NIR regions; (iii) therapeutic efficiency *in vitro* and *in vivo* with reduced side effects; (iv) potential use as a theranostic agent by obtaining second near-infrared (NIR-II) fluorescence images.

The incorporation of porphyrins in nanostructures is also recurrently reported in the literature to improve these first- and second-generation PSs, which, due to their several shortcomings that make their therapeutic efficacy unfeasible in their single form, when encapsulated, improve many properties related to their phototherapeutic effects.

As an example of a nanosystem that incorporates porphyrins, Shang *et al.* [153] reported a new theranostic nanomaterial targeting mitochondria called “PTPF-MitP”, by combining three functional groups: a lipocationic selective peptide as a mitochondrial targeting unit (MitP), *meso*-tetra(4-carboxyphenyl)porphyrin (TCPP) as a NIR fluorescent signaling unit, and platinum nanoparticles functionalized with polydopamine and TCPP as photothermal and PDT agents, respectively. In A549 human lung adenocarcinoma cells, the nanomaterial was in the mitochondria, proving the usefulness of the MitP unit in its targeting. In addition, the authors showed that this targeting was essential for the best therapeutic activity since nanomaterials not functionalized with MitP saw their photocytotoxicity reduced by half under 650 nm laser irradiation. Thus, its improved cytotoxicity, the fact that the death pathway adopted after photodynamic treatment is apoptotic given the release of cytochrome C, the increase in temperature observed after irradiation, and its visualization in real-time by imaging encourage the use of this “nanomissile” as a theranostic agent for lung adenocarcinoma.

**Figure 7:**
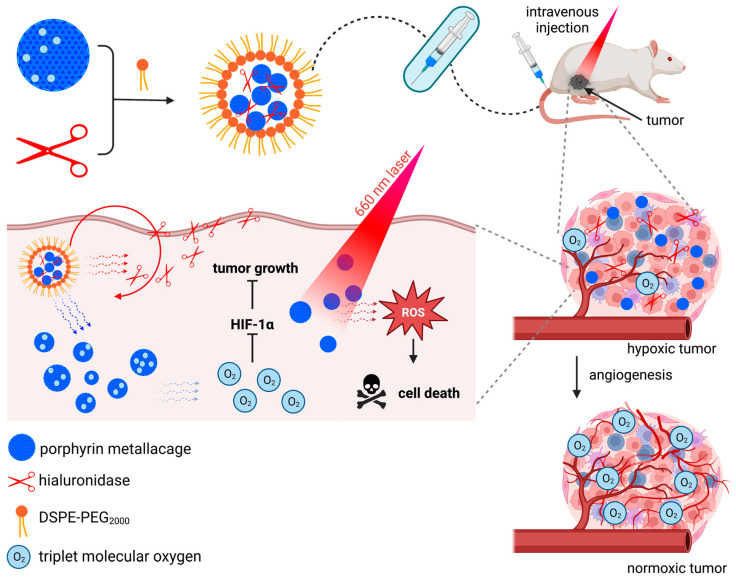
Formulation and mechanism of action of porphyrin metallacages nanoparticles containing hyaluronidase against hypoxic tumors reported by Jiao *et al.* [154]. The nanocarrier was prepared by combining a porphyrin metallacage, in which cavities are concentrated with triplet molecular oxygen, and the enzyme hyaluronidase, coated with 1,2-distearoyl-*sn*-glycero-3-phosphoethanolamine-*N*-(methoxy-polyethylene glycol)-2000 (DSPE-PEG_2000_). Hyaluronidase allowed the degradation of the extracellular matrix, resulting in greater formulation accumulation inside tumor cells. Molecular oxygen release from metallacages combined with hypoxia relieved by angiogenesis suppresses the expression of hypoxia-inducible factor-1α (HIF-1α) and consequent tumor growth inhibition. Irradiation with a laser emitting at 660 nm activates porphyrin, which, once activated, produces singlet oxygen, culminating in cell death.

The conception of porphyrin-containing nanoparticles may also contribute, for example, to better cell permeation and increased efficiency in a tumor environment poor in triplet molecular oxygen. This is proof of the study by Jiao *et al.* [154], in which they couple porphyrin metallacages capable of concentrating triplet molecular oxygen to proteolytic enzymes to break down hyaluronic acid (HA) moieties in an amphiphilic polymeric nanostructure 1,2-distearoyl-*sn*-glycero-3-phosphoethanolamine-*N*-(methoxy-polyethylene glycol)-2000 (DSPE-PEG_2000_; Figure 7). In this way, the incorporation of proteases allowed better permeation of the drug in cancer tissues since HA is overexpressed in tumor cells and is one of the main components of the extracellular matrix [155]; after irradiation at 660 nm, porphyrin was capable of producing singlet oxygen more proficiently than porphyrin in the free state, and the assembly of amphiphilic molecules made it possible to overcome the hydrophobicity of the PS candidate.

Dozens of other highly innovative strategies have been recently reported within the scope of porphyrin encapsulation, such as the design of triple action nanostructures (PDT, chemotherapy, and immunotherapy) [156], the combination of graphene quantum dots given their recognized photostability, biocompatibility, and minimal toxicity [157], as well as a vast array of techniques that make it possible to complement the therapeutic branch with prevention and diagnosis in the design of new and increasingly attractive porphyrin-based theranostic methods [158,159,160].

##### Porphyrazines

Porphyrazines are synthetic derivatives of the porphyrin class, which, instead of the typical methine carbons, have nitrogen atoms in their structural places. Although less known than porphyrins in their phototherapeutic application, these derivatives already have well-defined synthesis pathways and a wide range of structures associated with this family of dyes [161,162]. Like porphyrins, porphyrazines also have the ability to absorb light at specific wavelengths and generate ROS after light activation, which can lead to the destruction of cancer cells [163].

The cellular location of new photosensitizing molecules is usually one of the focuses of investigation since they should accumulate in organelles of high significance for cellular integrity and maintenance, guaranteeing the occurrence of cell death after light activation. The most desired target is usually the mitochondria, since this is crucial for the cell’s energy metabolism, and variations in the permeability of its membrane can trigger the release of cytochrome C and the consequent occurrence of the apoptotic death mechanism [164,165]. Despite this, studies prove that other organelles, including the Golgi apparatus, lysosomes, peroxisomes, and the cytosol, can be sites where photosensitization successfully occurs [166,167]. Additionally, demonstrating that mitochondrial uptake is not necessary for photodynamic success, in 2021, Tasso *et al.* [168] synthesized a series of five magnesium porphyrazine complexes in which photobleaching inside cellular plasmatic membranes occurs through an electron abstraction of the lipid double bond, resulting in irreversible damage to the membrane. Interestingly, they observed that the higher the PS photobleaching rate, the faster the membrane leakage was induced. This research also challenges the commonly held belief that photostability is a crucial requirement when developing new PSs and suggests that a defined photobleaching rate can be beneficial for developing dosimetry in hypoxic target regions and drug elimination.

A particularly studied subclass of porphyrazines is the cyanoarylporphyrazines, whose researchers have been introducing different aryl groups and complexing them with various metals, as is the example of the study by Yuzhakova *et al.* [169]. They prepared two cyanoporphyrazines complexed with gadolinium, differing in the side chains: one functionalized with fluorobenzene and the other with (benzyloxy)benzene. *In vivo*, both dyes showed intense fluorescence in tumor regions compared to healthy tissues, indicating selective accumulation. The authors also showed that these porphyrazines have promise as contrast agents and can be used in magnetic resonance imaging. Despite being interesting from a diagnostic point of view, only the (benzyloxy)benzene derivative caused moderate tumor growth inhibition. Complexation with iron of other analogs of tetracyanoporphyrazines [170], this time functionalized with naphthyl groups, despite seeing their cellular uptake reduced, proves to have distinct photodynamic interest since much higher photodynamic indices were observed for the metal-complexed dye. In addition to their interest as potential PSs, the authors show that they may serve as possible fluorescent probes for determining the viscosity of solutions [171]. Other recent studies report other aryl groups that potentiate this subclass of porphyrazines [166,172].

The encapsulation of porphyrazines has also been the subject of study [173,174], one of the most attractive studies recently reported being that of Mlynarczyk *et al.* [175]. These authors report the synthesis of a *seco*-porphyrazine, a derivative possessing one of the pyrrole units opened by oxidative processes. These derivatives are little studied due to their complex synthesis and isolation, as well as their high chemical instability. However, once obtained with high purity, it was studied for its potential in PDT in CAL27 and HSC-3 oral squamous cell carcinoma cells and HeLa cervical epithelial adenocarcinoma cells. With distinct phototherapeutic activity in its free form, it was encapsulated in different liposomes made up of four types of lipids: phosphatidylcholine (POPC), dioleoylphosphatidylethanolamine (DOPE), phosphatidylglycerol (PG), and 1,2-dioleoyl-3-trimethylammonium-propane chloride (DOTAP). Mixtures of DOTAP:POPC or PG:DOPE lipids showed more attractive effects than PG:POPC or DOTAP:DOPE, with researchers demonstrating that the lipid constitution of these nanostructures can condition the photoactivity of the compounds internalized in them.

Assigning a particular favorable characteristic to the phototherapeutic application can be challenging, generally requiring a large set of molecules so that it can be confidently stated that the introduction of a specific functional group confers a particular property. Yagodin *et al.* [176], in their recent study and based on all their knowledge about porphyrin-based molecules, have prepared a tetraquinoxalineporphyrazine that has attractive photodynamic properties: maximum absorption at wavelengths around 760–770 nm, improved stability compared to other derivatives, and a high relative singlet oxygen quantum yield. The authors explain that the bathochromic shift is justified by the π-extension of the quinoxaline group directly linked to the porphyrazinic ring, that light stability is conferred particularly by pyrazine, and that the eight anionic carboxylate substituents explain its increased water solubility. Based on the well-known Warburg effect, which describes the efficient uptake of glucose by tumor tissues to produce energy *via* glycolysis, several authors, including Klein and Ziegler [163], prepared candidates for glycoconjugate PSs to increase the uptake of these molecules by the tumor cells, “deceiving” them. Despite exhibiting appealing properties, their biological activity has not been evaluated.

##### Chlorins

Chlorins, also known as dihydroporphyrins, are porphyrinic dyes with attractive photophysical characteristics, namely improved absorption in the red region compared to traditional porphyrins [177]. The preparation of these molecules is usually initiated by derivatizing porphyrins; however, their purification is hampered by the fact that they are critically unstable, either because of the ease of retro-oxidation in porphyrins or because they are not very stable to light, as well as because, generally, multi-step synthesis is necessary, making their preparation even more challenging [178].

Clinically, chlorins already play a role, some of the most important being Temoporfin, Verteporfin, and Radachlorin. These commercial molecules are still a source of inspiration for creating new chlorins with improved properties and developing formulations that can improve them therapeutically or promote their theranostic application. Chlorophyll itself, a photosynthetic pigment present in the chloroplasts of plants and some algae, is used as a starting point for the synthesis of various chlorins [179,180], namely the well-known and recognized chlorin-e6 [181].

**Figure 8:**
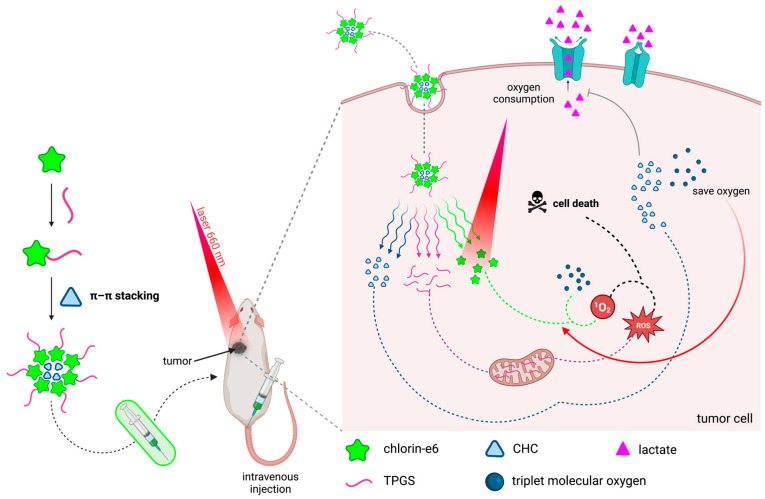
Preparation and mechanism of action of the lactate-aerobic respiration-inhibiting photodynamic therapy strategy suggested by Qin *et al.* [182]. The nanostructure was prepared by chemically conjugating chlorin-e6 with D-α-tocopherol polyethylene glycol 1000 succinate (TPGS) and, by hydrophobic π–π stacking interactions, α-cyano-4-hydroxycinnamate (CHC) units were co-assembled. Each of these elements was important for the therapeutic activity of the nanoparticle: CHC allowed the inhibition of lactate aerobic respiration, allowing the saving of triplet molecular oxygen required for the improved photodynamic activity of chlorin-e6, as well as TPGS, which, interacting with the mitochondrial complex II, induces reactive oxygen species (ROS) formation. These ROS, conjugated to singlet oxygen produced by chlorin-e6 activated after irradiation, culminate in cell death.

An example of this inspiration is, for example, the study by Qin *et al.* [182], where the researchers present a derivative of chlorin-e6, a PS whose hydrophobic nature and reduced activity in hypoxic environments strongly compromise its therapeutic action. Thus, based on these two shortcomings, Qin had two challenges that resulted in the preparation of a nanoplatform of undoubted significance: functionalizing structural chlorin with amphiphilic D-α-tocopherol polyethylene glycol 1000 succinate (TPGS) to modulate its hydrophobicity and, additionally, to induce the production of ROS by the interaction of TPGS with mitochondrial complex II, as well as its assembly into α-cyano-4-hydroxycinnamate units for the formulation of nanostructures capable of intervening in the regulation of lactate metabolism and therefore saving intracellular molecular oxygen (Figure 8). Another example that shows the relevance of working on already known PSs is the study by Kawasaki *et al.* [183], in which, through chlorin-e6 self-assembly in polymeric polysaccharides from maltotriose units, they prepared a PS system 780 times more potent than Photofrin. However, a disadvantage of chlorins, and most classes of PSs, is their poor targeting. As such, in a study of extreme significance, Yang *et al.* [184] developed chlorin-e6-loaded silica nanoparticles and, to specifically target gastric cancer cells, covered them with a cell membrane derived from cells of the same disease, obtaining excellent results both *in vitro* and *in vivo*. Other strategies have been adopted, such as integrating chlorins in maltotriose-functionalized nanoparticles [185] and HA-based carbon nanotubes [186].

The conjugation of dyes with molecules used as chemotherapeutic agents in a fusion approach of PDT with chemotherapy is a therapeutic strategy called chemophotodynamic therapy (chemoPDT). As an example, one of the most recent studies addressing this subject is that of Yang *et al.* [187], in which chondroitin sulfate-based nanoparticles co-charged with chlorin-e6 and paclitaxel are prepared. These nanosystems showed efficient photodynamic and chemotherapeutic synergy *in vivo*, releasing paclitaxel after the destruction of the nanostructure by laser irradiation for chlorin activation.

Structurally, several adaptations in the core of chlorins have been carried out, from the preparation of new pyrazolopyridine-bearing diphenyl chlorins designed from molecules with very relevant theranostic properties [178,188], their functionalization with fatty acids such as myristic acid to increase their cellular permeability [189], the introduction of groups with sulfur atoms such as thiophenes in order to improve their ability to form singlet oxygen [190], to their structural conjugation with sugar molecules and other compounds of biological interest [191,192].

##### Bacteriochlorins

Plant photosynthesis mainly involves chlorophylls, which have been extensively studied due to their spectroscopic and photophysical properties [193,194,195]. However, bacteriochlorophylls, natural bacteriochlorins found in some photosynthetic bacteria species, are also relevant in understanding fundamental processes and potential applications [196,197]. Structurally, bacteriochlorins differ from chlorins as they present two reduced pyrroles on opposite sides of the porphyrin-derived macrocycle (Figure 6).

Bacterichlorins can absorb electronic radiation in the visible to NIR region of 700 to 900 nm, penetrating deep into tissues [198]. While naturally occurring metal complexes derived from bacteriochlorophylls are unstable and have limited use, there are now synthetic compounds recently described that belong to the bacteriochlorin family and are highly photostable [199,200]. Therefore, two significant issues with bacteriochlorophylls must be addressed for their phototherapeutic application: firstly, they are prone to instability, which can lead to unintended dehydrogenation and subsequently the formation of a chlorin; and secondly, the rigidity of their macrocycle makes it challenging to perform synthetic transformations [198,201,202].

In the recent literature, one of the most reported strategies is the functionalization of bacteriochlorins with naphthalimides since these last molecules have an excellent ability to fluoresce, are endowed with good light stability, and have high Stokes shifts. Morozova *et al.* [203] showed that this conjugation resulted in the selective accumulation of malignant cells from murine tumor cells of S37 sarcoma, that excitation at the maximum wavelengths of the bacteriochlorin unit resulted in activity as high as that of individual bacterio-chlorin, and that the additional excitation of the naphthalamide unit results in increased therapeutic efficiency due to the transfer of energy from the fluorophore to the PS. In addition, the fluorescence properties of the fluorophore unit enhance the use of these molecules for theranostics. Other similar studies based on naphtalimide-bearing bacteriochlorins are also reported in recent original articles [202,204,205].

**Figure 9:**
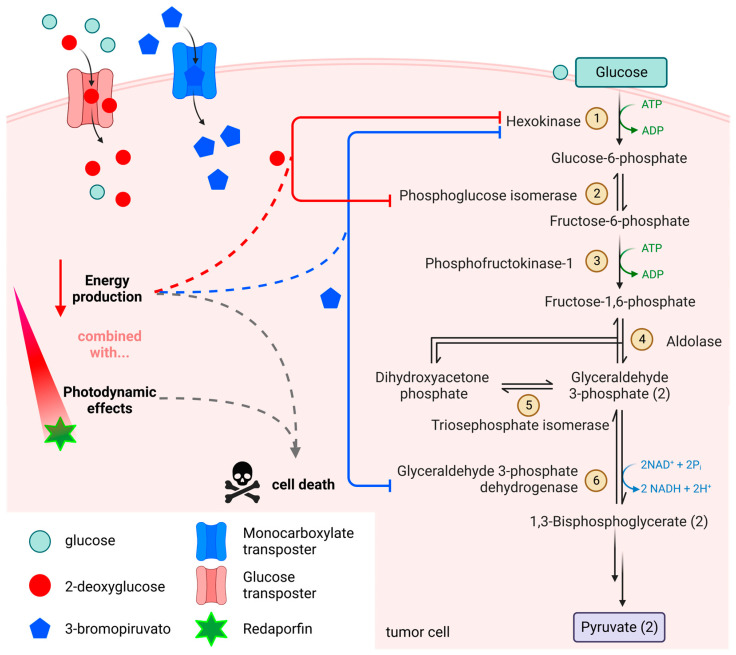
Role of glycolysis inhibitors combined with the photodynamic activity of Redaporfin in developing an efficient antitumor strategy proposed by Mendes and Arnaut [208]. 2-Deoxyglucose acts as an inhibitor of hexokinase and phosphoglucose isomerase, while 3-bromopiruvato is uptaken by cells through monocarboxylate transporters, which are overexpressed in tumor cells, targeting hexokinase and glyceraldehyde-3-phosphate dehydrogenase enzymes, both of which are decisive for the glycolytic pathway’s regulation. The energy imbalance due to the glycolysis blockade, mainly triggered by 3-bromopyruvate conjugated with the photosensitizer’s production of singlet oxygen, led to cell death.

Current research is focused on developing new therapeutic strategies using Redaporfin and understanding its mechanism of action. Studies by Lobo *et al.* have shown that low doses of light-activated PS can destroy the primary tumor and reduce the formation of metastases while also triggering antitumor immune responses in mice with CT26 tumors [206]. In addition, Karwicka *et al.* found that depriving the tumor of blood supply using this PS molecule led to a highly effective antineoplastic response *in vivo*, with tumors not reappearing in 67% of mice after more than a year [207]. Lastly, Mendes and Arnaut explored the potential of combining Redaporfin with glycolysis inhibitors 3-bromopyruvate and 2-deoxyglucose [208] (Figure 9). The authors explain that glucose transporters serve cells to absorb glucose and 2-deoxyglucose, which is analogous to glucose but cannot be metabolized. 2-Deoxyglucose acts as an inhibitor of hexokinase and phosphoglucose isomerase. Meanwhile, 3-bromopiruvato is taken up by cells through monocarboxylate transporters, which are overexpressed in tumor cells, inhibiting hexokinase and glyceraldehyde-3-phosphate dehydrogenase, crucial enzymes in the glycolytic pathway. The combined action, particularly of 3-bromopyruvate, with the excellent photobiological properties of the PS, studied in mice with large subcutaneous CT26 tumors, showed cure rates that were 22% higher than using Redaporfin alone and 33% higher than using only the glycolysis inhibitor.

Conjugation with chemotherapeutics such as erlotinib [209], the introduction of groups that allow improvements in their biocompatibility through hydrophilicity enhancement (e.g., Trizma and spermine groups [210,211]), and the functionalization of bacteriochlorins of natural origin or the design of new bioinspired bacteriochlorins [199,212], are other approaches described in recent works that deserve the scientific community’s attention.

In the field of oncology, and among the previously mentioned works, the study by Cheruku *et al.* is one of the most attractive. The authors prepared a series of chlorophyll-a and bacteriochlorophyll-a derivatives structurally conjugated with the erlotinib chemotherapeutic agent in different positions of the macrocycles through linking groups of different nature, having concluded that these two latter factors interestingly conditioned in a significant way their *in vivo* tumor selectivity and therapeutic effects.

##### Phthalocyanines

Phthalocyanines (Pcs) are porphyrin-based dyes composed of four isoindole nuclei joined by four nitrogen atoms. Among the porphyrinic PSs, Pcs may have been most recently studied due to their superior photochemical and photophysical properties [213]. Compared to conventional porphyrins such as Photofrin and Foscan, for which treatments are typically associated with skin photosensitivity, the fact that Pcs exhibit strong and sharp absorption wavelengths in the range of 650 to 800 nm causes this to be minimized, allowing for better light penetration into tissues and consequently the treatment of deeper pathologies [214]. Highlighting this class as potential PSs, several Pcs and their formulations are in clinical trials, namely the aluminum-complexed Photosens [215] and the liposome-loaded ZnPc “CGP55847” formulation [216].

Photosens sees an improvement compared to most Pcs in its biocompatibility. Structurally, this property is explained by the sulfonic groups, which indicated neurotoxicity in *in vivo* studies for tetrasubstituted dyes. Interestingly, the sulfonic groups’ number reduction contributed to filling this gap, with those disubstituted with sulfonic groups in opposite positions showing greater effectiveness. Phthalocyanines with sulfonyl groups are still studied nowadays [217].

Summarizing several examples of the structural changes made over the last few years, Table 3 gives a general overview of their influence on the therapeutic potential of Pcs.

The mediation of the therapeutic activity of PSs by other molecules of biological interest is also reported in the literature as an approach that significantly increases their cytotoxicity. Two of the most recent examples in the literature involve biomolecules, such as catechins [229] and cannabidiol [230], and chemotherapeutic agents, for example, doxorubicin [231] and dacarbazine [232].

Within the scope of the use of biomolecules, Nkune *et al.* [230] report the synthesis of a tetra 2-mercaptoacetate, ZnPc, that accumulates into the lysosome and mitochondria of A375 human metastatic melanoma cells, which, combined with cannabidiol, proved to be lethal for the cells under investigation, reducing cell viability by about 20%. This value becomes interesting since the photosensitizing agent, by itself, when irradiated, like cannabidiol, decreases only 50% of cell viability under the same experimental conditions.

Concerning chemoPDT, Doustvandi *et al.* [231] demonstrated that by combining doxorubicin and ZnPc and applying low doses of radiation, synergistic effects occur: death by autophagy and apoptosis, cell cycle arrest in G2/M, and a significant reduction in cell migration capacity were some of the phenomena observed with a higher incidence compared to treatments with a PS or chemotherapeutic agent separately.

Researchers have also focused on the encapsulation of Pcs in drug delivery systems, such as micelles [233], liposomes [234], nanocapsules [235], nanoemulsions [236], and, interestingly, in “nanodiamonds” [237,238].

These so-called “nanodiamonds”, as they contain several functional groups on the surface, such as amine-, amide-, hydroxyl-, carbonyl-, and carboxyl-groups, some of which are not present in ordinary graphene quantum dots, facilitate the binding of potential PSs. In 2019, Matshitse *et al.* [237] published a study involving nanodiamonds covalently bonded to boron-dipyrromethenes (BODIPYs) by amide bonds and stacked on a ZnPc derivative. This nanodiamond structure saw a very significant increase in its singlet oxygen quantum yield compared to individual molecules. Evaluated against MCF-7 cells, the nanodiamond bearing both PSs showed harmlessness in the dark and high photocytotoxicity. However, modest differences from nanodiamonds functionalized exclusively with BODIPYs or ZnPc were witnessed. Three years later, the same investigation group presented a new study evaluating benzothiazole-substituted ZnPcs with zero, three, or four positive charges linked or not covalently to the nanodiamonds [238]. The asymmetry and cationic character resulted in superior PDT activity.

Finally, another strategy of sublime significance that has been used to improve targeting is the loading of nanostructures with specific biomolecules, such as lipid carriers [239] and titanium dioxide nanoparticles [240], with folic acid, explained by the difference in expression of its receptors between normal cells and tumor cells, in which they are overexpressed, namely in prostate, breast, and ovarian tumors.

#### 4.1.2. Cyanine-Based Photosensitizers

Cyanines are a class of photosensitizing dyes consisting of two heterocyclic rings containing carbon atoms and at least one nitrogen atom, which are connected by a polymethine chain comprising an odd number of carbon atoms necessary for the resonance of the lone pair of electrons between the two nitrogen atoms of heterocyclic rings [241,242].

Although they are structurally simpler than molecules derived from porphyrin-based dyes, a wide range of molecules can be built from their skeleton. In their design, there are several constraints to consider for them to perform a potential phototherapeutic activity, namely:

- Polymethine chain length: The number of carbons present in the polymethine chain is the property that defines which class cyanines belong to. Cyanine-based compounds with a single carbon in the methine chain are called monomethine cyanines (Cy1), with three trimethines (Cy3), five pentamethines (Cy5), and seven heptamethine cyanines (Cy7). Cy5 and Cy7 are the most noted in the literature in the study of their photobiology properties since they efficiently absorb at higher wavelengths: while Cy1 absorbs at around 400 nm, Cy7 sees its maximum absorption peaks around 800 nm [243];
- Heterocycle units: For PDT purposes, the preparation of cyanines bearing heterocycles derived from indolenine, benzoindole, benzothiazole, benzoselenazole, benzoxazole, and quinolines is the most common. Among those mentioned above, indolenine and benz[*e*]indole derivatives are the most highlighted in the recent literature. Furthermore, anthracene units serving as heterocyclic units have been reported [244,245]. The nature of the heterocycles may condition the biological activity of cyanines, varying, for example, the stability of dyes to light [246];
- Central ring: The introduction of cyclic structures in the center of cyanines is a strategy that allows for improving the rigidity and chemical stability of this class of molecules. It is especially common in the case of Cy5, where there are rings with four or five members (squaraine and croconaine dyes, respectively [247,248]) and, in the case of Cy7, cyclohexane rings or their derivatives (for example, a boron fluoride complex within the core structure [128]). The functionalization of Cy7’s cyclohexane ring with halogenated atoms such as bromine and chloride is common [249], which serve as excellent leaving groups for functionalization with other groups of interest [250,251,252];
- *N*-alkyl chains: Since cyanines are intrinsically lipophilic compounds, this characteristic can be modulated by increasing or decreasing the length of *N*-aliphatic chains. This modulation of biocompatibility is also carried out, for example, by introducing sulfonic groups [249,252], carboxylic acids [253,254], pyridines [255], or even more attractively, by functionalizing them with PEG chains [253] or HA units [256]. The conjugation of cyanines with porphyrin-based dyes, adding them *via N*-alkyl chains, is another recently reported strategy to formulate new promising PSs [257]. A further attractive approach is the dimerization of these dyes, building molecules with two covalently linked cyanine nuclei, these chains serving as bridges between units [258]. The targeting of these compounds can also be enhanced, for example, by introducing specific antibodies to these chains [259];
- Halogenation and the so-called “heavy atom effect”: The introduction of halogens and other heavy atoms, such as selenium [260,261], contributes above all to more efficient anticancer activity and, by increasing the lifetime of these dyes in the triplet state, to the improvement of their singlet oxygen production. The presence of iodine atoms is highlighted as the form of halogenation that most actively increases therapeutic activity [249,254,262]. Interestingly, Semenova *et al.* provide evidence that, effectively, there is no linearity between the therapeutic effects and the number of iodine atoms introduced, reporting that the “ideal” number for the Cy7 they prepared is two iodine atoms [263]. Despite this, for indolenine-based Cy5, bromination proved to be more advantageous [264]. However, for benz[*e*]indole-based brominated Cy5, the same research group shows that there is no advantage in these halogenated ones compared to non-halogenated ones, indicating that the heterocycle may play a role [265]. Intending to improve its bioavailability, Shi *et al.* introduce the trifluoromethyl group into the Cy7 *N*-alkyl chains, stating that this structural modification can improve the cellular uptake of the dye [266].

**Figure 10:**
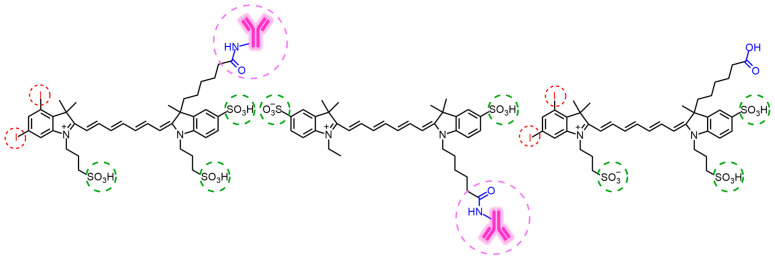
Trastuzumab antibody-functionalized iodinated and non-iodinated heptacyanines prepared by Kobzev *et al.* [259] for photoimmunotherapy. The structural combination with the antibody (pink) contributed to the immunotherapeutic activity of the dyes; the sulfonyl groups (green) made the cyanines more biocompatible; and the iodine atoms (red) allowed an increase in the production of singlet oxygen.

Concerning cyanines, two very recent works should be highlighted: the first in which the authors take advantage of the fact that the prepared Cy3 forms J-aggregates in a strategy that serves to enhance therapeutic efficacy [267], and the second in which analogous iodinated and non-iodinated cyanines with an antibody covalently joined to them through an amide linker are synthesized to develop a methodology that also allows simultaneous treatment and monitoring of tumors (Figure 10) [259].

In the first work, Li *et al.* [267] prepared an iodo-indolenine- and quinoline-based Cy3, which, in its free form, has maximum absorption at 630 nm. However, as soon as it is administered intravenously, the dye aggregates under the control of anions with maximum absorption at 700 nm and is directed towards the tumor *via* the enhanced permeability and retention (EPR) effect, a mechanism in which macromolecular compounds can accumulate progressively in the vascularized cancer area. In an intracellular environment, RNA molecules constituted the negatively charged microenvironment for reconstructing these aggregates, accumulating preferentially and gradually in the nucleoli of tumor cells. The authors predict that this “smart J-aggregation” can be used in future studies since it integrates the ability of tumor targeting and improves the therapeutic efficacy of the potential PS molecule.

In the second study, Kobzev *et al.* [259] combine PDT with immunotherapy through the design of new photoimmunotherapeutic agents resulting from the conjugation of Cy7s, iodinated and non-iodinated, with the antibody trastuzumab (Figure 10). The iodinated conjugate demonstrates a greater capacity to produce singlet oxygen, as expected according to the effect of the heavy atom. The non-iodinated conjugate did not show significant phototoxicity under 730 nm light-emitting diode (LED) irradiation, acting only as an immunotherapeutic agent. In contrast, the iodinated one, in addition to exhibiting intense fluorescence signals that allow monitoring of the conjugate’s distribution in the body, suppresses tumor growth about five times. The immunotherapeutic effect is demonstrated when comparing tumor suppression with the dye without antibody conjugation, which only suppresses about 1.4 times. With such impressive results, the researchers believe that PS-antibody conjugation could be a tool further explored in other cyanine-based PSs.

Incorporating central rings in Cy5 is also common in preparing squaraine dyes, with a four-membered central ring derived from squaric acid, and croconaine dyes, with a five-membered ring derivative from croconic acid. Between these two sub-classes, squaraines are the most discussed in the recent literature, showing interesting photoproperties. Furthermore, compared to Cy5 with conventional methine chains, these structures, as they contain carbon rings, are known to be more chemically stable.

Regarding the photodynamic activity of squaraines, our last review focuses on the effect of the various structural modifications carried out on them on their biological activity [247]. Nonetheless, it should be noted that the main structural changes carried out more recently in these molecules are mostly the replacement of one of the oxygen atoms of the four-membered central ring by amines (for example, ethanolamines, picolylamines, and sulfonamides) [268,269,270] and the functionalization of that same ring with barbituric acid derivatives [271].

Since their debut in 1970, croconaine dyes have been mentioned in the scientific and patent literature [248]. However, the level of research conducted on croconaine dyes has not matched that of squaraines. Croconaines can absorb in the NIR region at higher wavelengths than squaraines and exhibit enhanced fluorescence properties, making them outstanding for biomedical applications [272]. As such, they are described in the recent literature as extraordinary molecules with potential use in diagnostic techniques (for example, brain tumors [273]), as well as in PDT approaches and photothermal [274,275] and, consequently, as theranostic agents [272].

**Figure 11:**
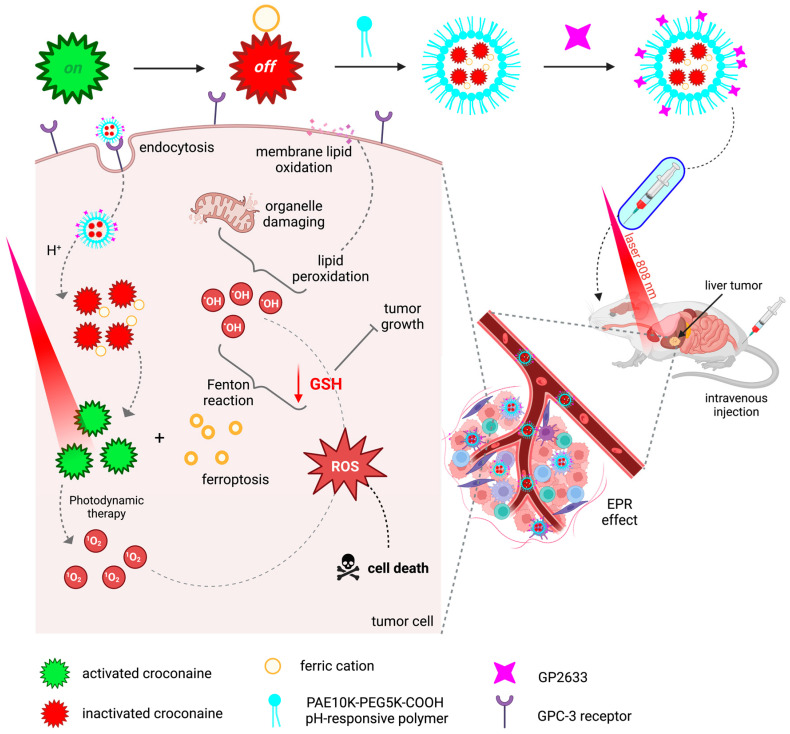
Synthesis and mechanism of action of the “on/off” croconaine-containing nanoparticle reported by Sun *et al.* [276]. They created a croconaine dye that exhibits absorption capacity in the near-infrared range and pH-responsive NIR-II fluorescence (“on”) that complexed with ferric ions and conducted its absorption loss, fluorescence ability, and photocytotoxicity (“off”). The dye-ferric complex (“off”) was then encapsulated with a pH-responsive polymer connected to the GP2633 glypican-3 (GPC-3) receptor-specific peptide. Through the “enhanced permeability and retention” effect (EPR), the nanoparticles were endocytosed by GPC-3 receptors. The proton-rich microenvironment led both the polymer degradation and the ferric-dye coordination bonds to break, activating its second near-infrared fluorescence (“on”). Singlet oxygen was generated after exposing the croconaine dye to 808 nm laser irradiation. The ferric cations released induced ferroptosis, were reduced to ferrous by consuming glutathione (GSH), and consequently inhibited tumor growth. Ferrous ions promote the production of cytotoxic hydroxyl radicals through Fenton’s reaction.

An example of work that validates the theranostic potential of croconaines by addressing their photodynamic activity is that of Sun *et al.* [276] (Figure 11). Because there are few amphiphilic PSs with absorption capacity in the NIR range and pH-responsive NIR-II fluorescence, the researchers combined all these properties in a croconaine dye. The dye was coordinated with ferric ions, resulting in its absorption loss, fluorescence ability, and photocytotoxicity. It was subsequently encapsulated with a pH-responsive polymer connected to the glypican-3 (GPC-3) receptor-specific peptide GP2633. By EPR, croconaine-containing nanoparticles were routed to the target tissue and endocytosed by GPC-3 receptors. Given the acidic pH characteristic of the tumor microenvironment, the polymer was degraded and the coordination bonds were broken, causing its NIR-II fluorescence activation. After 808 nm laser irradiation, the croconaine showed singlet oxygen generation ability. The released ferric cations induce ferroptosis as, in addition to being used for nuclear magnetic resonance imaging, they are reduced to ferrous by the consumption of GSH, inhibiting tumor growth, and through Fenton’s reaction, the production of cytotoxic hydroxyl radicals is promoted.

#### 4.1.3. Phenothiazine-Based Photosensitizers

Phenothiazines are dyes with a central thiazine core and two benzene rings condensed to the thiazine ring. Given their biological, photochemical, and photophysical qualities [277,278], these dyes have recently been studied as PSs, more focused on the inactivation of bacterial, viral, and parasite strains [279,280,281,282]. Two of the best-known phenothiazines are methylene blue and toluidine blue O, with methylene blue recurrently reported in clinical cases in medical approaches to the treatment of bacterial infections, including for dental diseases, proving to be effective, safe, and with interesting aesthetic results [280,283]. In addition, derivatives of methylene blue, that is, 3,7-disubstituted dyes of the phenothiazine nucleus, have been synthesized, functionalizing these positions with several amines to be studied for photobiological and optoelectronic applications (for example, as sensitizers of organic solar cells and for the construction of dye-based lasers) [278]. Inspired by these molecules’ core, sonodynamic therapy agents have been investigated [284].

Although less common than its antibacterial activity, some molecules derived from phenothiazines are still created within the scope of their application in cancer PDT. One of the most attractive examples is that of Yang *et al.* [285], where they conjugate methylene blue with camptothecin through an activatable linker that contains a disulfide bond susceptible to breakage by GSH. Administered *in vivo*, the authors did not observe physiological toxicity, suggesting safety and biocompatibility, making them believe that they created a molecule capable of overcoming the limitations of methylene blue and camptothecin in their individuality.

#### 4.1.4. Boron-Dipyrromethene-Based Photosensitizers

In recent years, research has significantly increased on the 4,4-difluoro-4-bora-3a,4a-diaza-s-indacene dyes, boron-dipyrromethenes, or BODIPYs. This has resulted in a notable rise in publications related to this chromophore core, showing its high versatility concerning its technological [286,287] and biomedical applications [99,288,289,290]. Within the scope of its phototherapeutic application, it is important to emphasize that BODIPYs offer a viable alternative to porphyrin-based compounds, owing to their flexibility in synthesis and photochemical properties [291], and consequently exhibit unique properties when modified and functionalized (Table 4).

One of the most attractive and vaguely reported approaches is the creation of structures made up of BODIPY PS units designed by self-assembly directed by the coordination of metallic ions, such as platinum and ruthenium, called supramolecular coordination complexes. These complexes can exhibit various shapes, including prisms, rectangles, hexagons, and triangles, among others, with the geometry they adopt highly influencing their photodynamic success [319,320]. Furthermore, the fact that they are coordinated prevents their aggregation and, consequently, their more efficient generation of ROS, and given the metal “heavy atom effect”, singlet oxygen production is also augmented [321]. Additionally, coordinating metals can also serve as chemotherapeutic agents, this being a chemoPDT modality [320].

There is nothing more thoughtful than taking the chemical and biological differences between tumor and healthy cells and, from these, building systems that allow their accumulation and activation only at the target site. Although not exclusive to BODIPYs, many “on/off” strategies, such as the one previously detailed on croconaines (Figure 11), have been extensively reported for this class of dyes [322,323]: the development of polymers, nanoparticles, or pH-responsive PSs that are only activated in acidic media characteristic of the tumor microenvironment [324,325]; the creation of GSH-cleavable quencher moieties containing PSs since the concentration of GSH in tumors is exacerbated higher than in normal cells [326,327]; and the design of systems that undergo activation by enzymatic degradation [328,329].

On the last topic, and by way of example, Juang *et al.* [329] took advantage of the fact that the level of tyrosinase, the enzyme that regulates melanin production through melanogenesis, is closely associated with melanoma severity by functionalizing a BODIPY dye with its inhibitor phenylthiourea to improve therapeutic efficacy against a tyrosinase-positive melanoma cell line. The authors verified an increase in its cellular uptake compared to a phenylthiourea-free compound, as well as the ability to produce ROS and significant effects on cell viability when irradiated. These results denote that this is a successful method and could be used to create even more efficient PSs for treating melanoma in the future.

## 5. Conclusions and Future Perspectives

Photodynamic therapy is a therapeutic strategy that, in recent years, has been growing exponentially in the most diverse areas that concern it to create new approaches that allow solving problems in today’s society, such as diseases of oncological origin. In this review, the authors intended to show how multidisciplinary PDT is, depending on knowledge about several scientific subjects, and whose combination is essential to its development and progression. Thus, based on this knowledge, we present the PDT basic principles from a clinical and photophysical-chemical point of view, showing how, theoretically, PS molecules act on the target tissue and what mechanisms they can adopt to produce cytotoxicity, the role of the three essential elements to improve phototherapeutic results, as well as the progression and alternatives that have been explored in recent years for the conception of improved therapeutic modalities to overcome the PDT weaknesses.

Regarding PSs, we emphasize that the slow progression of PDT is mainly due to the lack of clinically approved molecules, so the need to design new compounds that potentially exhibit properties inherent to those of an ideal PS is highly pertinent. As such, the concerns of current research in the preparation of new PSs are related to photophysical properties such as singlet oxygen production and absorption capacity at red and NIR wavelengths, modulation of lipophilicity inherent to most of the nuclei of these classes of compounds, as well as their direction to the precise location to be treated. To this end, approaches of high scientific interest have been created, with desirable results and elucidating how prominent the progress in oncology and medicine can be, especially when chemistry and nanotechnology subjects are merged.

Thus, given the colossal range of results that reinforce the proficiency of this therapeutic approach, there is a need to make “leads” the most relevant and best-founded based on encouraging results to be applied clinically soon. Furthermore, the development of new PS classes as well as the analysis of the therapeutic effects of the hitherto less explored ones, the creation of new delivery systems, and new targeting methods are equally desired since a more extensive and comprehensive spectrum of treatment methodologies will allow for a more successful fight against this very heterogeneous disease.

## Figures and Tables

**Table 1:** Comparison of various light sources used in photodynamic therapy and their main advantages and disadvantages.

Light Source	Description	References
**Sun light**	The use of sunlight as a treatment for dermatological conditions such as actinic keratosis is a cost-free and less painful option that can be easily performed at home. However, it is highly dependent on the weather conditions of the region, and there is no way to regulate the amount of energy emitted.	[88,89]
**Lamp lights**	Lamp lights are cheap, portable, easy to use, and emit at a wide range of wavelengths. In addition, filters eliminate radiation emitted without interest in the excitation of the photosensitizer, namely at short wavelengths. However, they have the disadvantage of losing energy in the form of heat, which is why they emit low-intensity light and are mainly limited to treating dermatological diseases.	[84]
**Light-emitting diodes (LEDs)**	LEDs are light sources with high power stability, capable of irradiating large areas, thermally non-destructive, inexpensive, small in size, and easily transportable. Combining lamps emitting at different wavelengths to excite several photosensitizers is possible with the same equipment. The light is incoherent and polychromatic, emitting in a narrow region of the electromagnetic spectrum.	[90,91]
**Lasers**	Lasers are currently the most commonly used clinically and produce high-intensity, coherent monochromatic light. The coherence contributes to the precise control of the fluence applied to the target tissue, which is more difficult to assess using incoherent sources. They can be coupled to optical fibers to deliver light to highly inaccessible tissues. It is the most expensive light source compared to those above. Comparative studies show comparable photodynamic efficacy using lasers or LEDs.	[78,84,92,93]

**Table 2:** Ideal properties of a photosensitizer (PS) for photodynamic therapy (PDT).

Property	Description	References
**Easy synthesis**	Optimizing PS synthesis is crucial for high yields and purity, affecting production scale and cost.	[3,113]
**Absorption in the visible and near-infrared regions**	Low-energy radiation reduces harm to healthy tissues and increases light permeability in biological tissues for deeper activation of PS.	
**Amphiphilicity**	Amphiphilic compounds are water-soluble and can easily cross the lipid membranes of cells, ensuring their availability and distribution, allowing the PS to target and accumulate in abnormal tissues more efficiently.	[123]
**Various routes of administration**	The PS or its formulation must allow safe and painless administration, whether performed orally, topically, or intravenously. In addition, several administration routes will enable the use of the same molecule in a broader range of diseases.	[124]
**Selective tumor accumulation**	It should be able to reach the neoplasia in a short time as well as selectively accumulate in tumor cells.	[125]
**Harmless in the absence of light**	Molecules that intrinsically have cytotoxicity should not be studied as potential PSs. To be applied in PDT, photosensitizing candidates must exhibit zero to low toxicity in the dark and significant cytotoxicity only when irradiated.	[123,126]
**Resistance to photobleaching**	The term “photobleaching” refers to the loss of the ability of the PS to absorb light due to its degradation during irradiation. Resistance to this phenomenon allows the use of higher energy light sources, which may result in deeper tissue penetration and greater therapeutic efficacy.	[127,128]
**Long-term** **triplet state lifetime**	The triplet state PS reacts with triplet molecular oxygen to produce ROS for type I and II PSs. Long-term triplet state lifetime enables a prolonged generation of cytotoxic species, which is crucial for the death of target cells.	[129]
**Rapid clearance from the body**	A rapidly cleared PS from the organism significantly decreases the duration of its presence in the body, thereby minimizing the risk of toxicity to healthy tissue and reducing the risk of side effects such as skin photosensitivity.	[126]

**Table 3:** Summary of the main structural modifications made to phthalocyanines (Pcs) in recent years, main aspects and conclusions that can be drawn from their photobiological effects.

Structural Variation	Specification	Main Aspects and Conclusions	References
**Metal coordination**	**Zn, In and GaPcs**	Various metals were introduced into benzimidazole tetrasubstituted Pcs. The In-complexed one showed highly efficient singlet oxygen generation and *in vitro* phototoxicity.	[218]
	**RuPcs**	Ruthenium complexation can create effective Pcs for PDT, known for its medicinal properties. In addition, loading NO^+^ and NO_2_^−^ units onto metal allows the release of nitric oxide, boosting light toxicity and causing selective effects seen *in vitro*.	[219,220]
**Presence of chalcogen atoms**	**O, S, Se**	Bathochromic shifts were observed for S and Se-bearing Pcs. The Se proved beneficial in increasing absorptivity and singlet oxygen production. PDT effects were observed for both derivatives, but the first derivative showed greater efficiency *in vitro*.	[221]
**Charges number**	**0, 4 or 8 positive charges**	Improved properties (singlet oxygen production, water solubility, and cellular uptake) were perceived for cationic compounds compared to neutral ones, and lower half-maximal inhibitory values were observed for eight times positively charged compounds for MCF-7 cells.	[222]
**Functionalization with biomolecules**	**Sugar units**	Ruthenium-complexed Pcs were functionalized with glucose, galactose, and mannose. Adding sugar did not enhance cellular uptake, but one-bearing mannose proved better for PDT use.	[223]
		Lactose Si-complexed Pcs proved to be biocompatible, stable in aqueous media, and efficient in ROS generation, showing *in vitro* high photocytotoxicity and selective tumor accumulation *in vivo*.	[224]
	**Biotin**	Biotin-PEG-bearing SiPc has been shown to selectively accumulate in tumor tissue and reduce biotin receptor-overexpressed tumor growth progression *in vivo*. In addition, PEG units made the potential PS more soluble in water and less prone to aggregation.	[225]
		It was perceived that self-assembled biotin-amine SiPc of different sizes (10, 20, 40, and 90 nm), depending on the percentage of surfactant, exhibited targeting and improved photoactivity *in vivo*, especially for the 20 nm particles after avidin-presence disassembly.	[226]
	**Glycyrrhetinic acid**	The interest in conjugating glycyrrhetinic acid to SiPc is due to its overexpressed receptors in liver cancer. The PS candidate was shown to effectively destroy the liver tumor tissue *via* necrosis and apoptosis. Side effects have not been observed *in vivo*.	[227]
	**Chalcones**	Chalcone-bearing cationic Zn- and In-complexed Pcs saw, for MCF-7 breast adenocarcinoma cells, half-maximal inhibitory concentration values reduced to less than half after irradiation. Despite its promise, mechanisms of action still need to be studied.	[228]

**Table 4:** Strategies recently reported on how to structurally improve or enhance certain aspects of boron-dipyrromethene (BODIPY)-based photosensitizers (PSs).

Aim	How?	Main Aspects and Conclusions	References
**Long-wavelength absorption**	**Conjugated double bonds and heavy atom introduction**	The length of π-conjugate bonds influences the ability to absorb at redshifted lengths, which is why authors have invested in the synthesis of thiophene- and phenyl-fused BODIPYs, in the introduction of pyrrole rings, and, alternatively, in heavy atoms.	[292,293,294]
**Improve singlet oxygen production ability**	**Halogenation**	The inefficient intersystem crossing (ISC) of BODIPYs limits their usefulness in PDT. As for most PS classes, halogenation is a way to improve this photophysical property. Introducing iodine atoms helps the ISC in a more pronounced way than bromination.	[295,296,297]
	**Organic metal complexes**	Aksakal *et al.* examined new ruthenium and iridium BODIPY complexes. They discovered that singlet oxygen generation increased by twenty times with ruthenium. Meanwhile, Jana *et al.* achieved quantum yields of 67% for a cobalt-complexed dye. These and other findings suggest a connection between these complexes and the singlet oxygen production ability.	[298,299,300]
	**Anthracene, pyrene, and fullerene conjugation**	Callaghan *et al.* studied several BODIPY dyes with diverse anthracenes and the pyrene group, all showing greater ISC than the phenyl group bearing one. Fullerene derivatives are also effective PSs, producing ROS efficiently with good biocompatibility and easy body clearance.	[301,302]
	**Sulfur core-fusion**	Thiophene-fused BODIPYs have shown improved singlet oxygen quantum yields (from 4% to 85%). Oscillations in its ability to carry out type II reactions were due to different functional groups introduced in its *meso* position.	[292]
	**BODIPY dimers and trimers**	Combining several BODIPY units covalently linked in the same structure can influence properties, namely the increase in the production capacity of singlet oxygen. This effect is shown by Lu *et al.* for dimeric dyes and by Prieto-Castañeda *et al.*, whose molecular geometry of analogous trimers is also shown to influence this photophysical property.	[303,304]
**Targeting specific organelles**	**Cationic character**	Cationic BODIPYs can become more hydrophilic and gain the ability to target mitochondria. They are attracted to the negatively charged inner mitochondrial membrane, accumulating in the mitochondrial matrix through a concentration gradient.	[305,306,307]
	**Morpholine group introduction**	The introduction of the morpholine into the core of BODIPYs induces their directing to the lysosome organelle. This finding was observed by several research groups in BODIPY dyes, as this group was introduced to the dyes through a linker.	[308,309,310]
	**Amphiphilicity and biomolecule mimetize**	Wang *et al.* have designed a phospholipid-mimetizing BODIPY-based fluorescent surfactant with two hydrophobic chains for cell membrane imaging and PDT. Concerning the latter, its effect is induced by damaging the tumor cells’ cytoplasmic membrane.	[311]
**Enhance biocompatibility**	**Aminoacid functionalization**	Amino acid conjugation is a practical approach to improving aqueous solubility and ameliorating their biocompatibility and photobiological efficacy. For example, the aspartic acid-modified BODIPY reported by Yu *et al.* exhibited enhanced aqueous solubility, singlet oxygen generation ability, and a good phototoxicity ratio.	[312]
	**Polymer-junction self-assembly**	To enhance the biocompatibility of BODIPYs, these molecules can be modified by attaching PEG derivatives or other hydrophilic polymers. This modification helps to reduce non-specific interactions with biomolecules and improves the stability of self-assembled BODIPY-based polymer-conjugated nanoparticles.	[306,313]
**Enhance tumor targeting**	**Polyamine chain introduction**	Tumor cells require amine growth factors and regularly exhibit polyamine transporter overexpression. This makes it possible for BODIPY-polyamine molecules to be transported to cancerous cells, which can improve the effectiveness of these tumor-targeting drugs. In addition, these transporters tolerate the uptake of diverse amines, making them a valuable tool for drug delivery.	[314]
	**Sugar-conjugate structures**	Recent research has revealed that galactose-containing macromolecules can successfully target various types of cancer. Combining lactose with BODIPYs efficiently targets and impacts tumor cells through enhanced recognition and interaction with overexpressed specific receptors.	[315,316]
		Glucose Transporter 1 is often overexpressed in cancer cells, increasing glucose intake and metabolism. As a result, glycosylated BODIPY triangular skeletons were designed by Durán-Sampedro *et al.* to promote cellular uptake through the Warburg effect.	[317]
	**Polysaccharide-conjugate structures**	For example, hyaluronic acid (HA) is commonly used as a polysaccharide-based drug carrier since it is water-soluble, has excellent biocompatibility, and is harmless. Chen *et al.* prepared an HA-BODIPY through an azide linker group that only triggered phototherapeutics inside tumor cells. This behavior is due to its self-assembled form in the extracellular environment; therefore, ROS production is inhibited.	[318]
